# Cardiovascular Problems in the Fragile X Premutation

**DOI:** 10.3389/fgene.2020.586910

**Published:** 2020-10-08

**Authors:** Nattaporn Tassanakijpanich, Jonathan Cohen, Rashelle Cohen, Uma N. Srivatsa, Randi J. Hagerman

**Affiliations:** ^1^UC Davis MIND Institute, UC Davis Health, Sacramento, CA, United States; ^2^Department of Pediatrics, Faculty of Medicine, Prince of Songkla University, Songkhla, Thailand; ^3^Fragile X Alliance Clinic, Genetic Clinics Australia, Melbourne, VIC, Australia; ^4^Division of Cardiovascular Medicine, Department of Internal Medicine, UC Davis Medical Center, Sacramento, CA, United States; ^5^Department of Pediatrics, University of California, Davis, Davis, School of Medicine, Sacramento, CA, United States

**Keywords:** fragile X, *FMR1*, premutation, cardiovascular, arrhythmia, autonomic dysfunction, hypertension

## Abstract

There is a dearth of information about cardiovascular problems in fragile X premutation carriers who have 55–200 CGG repeats in fragile X mental retardation 1 (*FMR1*) gene. The *FMR1* expansion in the premutation range leads to toxic RNA gain-of-function resulting in cellular dysregulation. The mechanism of RNA toxicity underlies all of the premutation disorders including fragile X-associated tremor/ataxia syndrome, fragile X-associated primary ovarian insufficiency, and fragile X-associated neuropsychiatric disorder. Cardiovascular problems particularly autonomic dysfunction, hypertension, and cardiac arrhythmias are not uncommon in premutation carriers. Some arterial problems and valvular heart diseases have also been reported. This article reviews cardiovascular problems in premutation carriers and discusses possible contributing mechanisms including RNA toxicity and mild fragile X mental retardation protein deficiency. Further research studies are needed in order to prove a direct association of the cardiovascular problems in fragile X premutation carriers because such knowledge will lead to better preventative treatment.

## Introduction

The Fragile X Mental Retardation 1 (*FMR1*) gene is located at Xq27.3 and the 5′-untranslated region contains a trinucleotide repeat of cytosine-guanine-guanine (CGG), that has a normal range of 5 to 45 repeats with a mean of 30 in the general population. The full mutation of the *FMR1* gene contains >200 CGG repeats and this causes fragile X syndrome (FXS), the most common inherited cause of intellectual disability and the most common single gene cause of autism spectrum disorder. This condition leads to silencing of *FMR1* gene by methylation and subsequent loss of its product, fragile X mental retardation protein (FMRP). FMRP is a messenger RNA (mRNA)-binding protein and regulates translation at the synapse of hundreds of mRNAs essential for synaptic plasticity. Therefore, its absence leads to significant cognitive and social impairment (Hagerman et al., 2017; Rajaratnam et al., 2017).

Moreover, cardiovascular problems including mitral valve prolapse, aortic root dilatation, and hypertension are commonly documented in individuals with FXS (Loehr et al., 1986; Waldstein and Hagerman, 1988; Sreeram et al., 1989; Utari et al., 2010; Ramírez-Cheyne et al., 2019). These problems were explained as a result of diminished or absent FMRP which leads to abnormal connective tissue structures including shortened and fragmented elastin fibers. However, cardiovascular conditions in premutation carriers, who have 55–200 CGG repeats on *FMR1* gene, are still rarely recognized except in occasional case studies (Riddle et al., 1998; Jacquemont et al., 2003; Coffey et al., 2008; Chonchaiya et al., 2010; Hunsaker et al., 2011; Hamlin et al., 2012). Since the premutation is common, 1 per 200 in women and 1 per 400 in men (Tassone et al., 2012b), the cardiovascular problems which can occur in carriers are relatively common. Our aim in this review is to advance understanding about possible risks and mechanisms of cardiovascular disorders in premutation carriers which will hopefully stimulate further research and treatment studies.

## The Fragile X Premutation

Unlike individuals with FXS who usually have remarkable clinical features including intellectual disability, autism spectrum disorder, macroorchidism, hyperactivity, prominent ears, and hyperextensible finger joints, the premutation carriers have subtle presentations. Medical problems which are increased in carriers compared to controls without the premutation include autoimmune diseases (Winarni et al., 2012), premature menopause before age 40 and fertility problems (fragile X-associated primary ovarian insufficiency; FXPOI) (Wheeler et al., 2014; Campbell et al., 2016), and the neurodegenerative disorder involving an intention tremor, ataxia and cognitive decline later in life called the fragile X-associated tremor/ataxia syndrome (FXTAS) (Jacquemont et al., 2004; Hagerman and Hagerman, 2016). FXPOI is seen in approximately 20% of female carriers and FXTAS is seen in 40% of male carriers and approximately 13% of female carriers. In addition, psychiatric problems are common, particularly anxiety, depression, obsessive-compulsive disorder, mood disorder, and they fall under the umbrella term of fragile X-associated neuropsychiatric disorders (FXAND). One or more of these psychiatric problems can affect up to 50% of carriers (Wheeler et al., 2014; Hagerman et al., 2018).

Cardiovascular problems in premutation carriers have been documented but most cases were mentioned indirectly as a part of case studies or reviews about FXTAS. Here we present case histories of three carriers who had significant cardiovascular conditions along with a review of the literature of each condition in carriers.

## Case Presentation

### Case 1

A 61-year-old female with a *FMR1* premutation allele of 76 CGG repeats who is physically active. She has two children with FXS, so they were the initial probands. Her father also had FXTAS and he had ischemic heart disease. She has hyperextensible joints and recurrent knee dislocation with skiing. She had a 4 year history of paroxysmal atrial fibrillation largely controlled with sotalol hydrochloride 40 mg twice daily. Whilst not aware of palpitations, she did note exertional breathlessness and slight limitation of her physical activity. She also noted pre-syncopal symptoms related to conversion pauses which would occur on average 2–3 times per week. Holter electrocardiogram monitoring confirmed her to have paroxysmal episodes of atrial fibrillation up to 7 h at a time, with significant conversion pauses of up to 2.3 s in length. This also demonstrated periods of rapid ventricular response with up to 130 bpm. Her exercise thallium scan result was normal, and her echocardiogram and cardiac computer tomography revealed trivial pericardial effusion. Laboratory panel results were unremarkable. Medications included atorvastatin 40 mg daily, aspirin 100 mg daily, sotalol hydrochloride 40 mg twice daily, denosumab 60 mg twice yearly.

Physical examination showed normal vital signs although often with an irregular pulse consistent with atrial fibrillation. Aside from a narrow face and slightly protuberant ears, there were no other stigmata associated with her fragile X premutation of 76 CGG repeats. Cardiovascular examination was normal.

Primarily in view of her symptomatic conversion pauses and refractoriness and intolerance to higher doses of medications, pulmonary vein isolation and atrial ablation were successfully performed. She remained in sinus rhythm and was discharged home on sotalol 40 mg twice daily and dabigatran 110 mg twice daily for 2 months and is followed in cardiology clinic.

#### Review of Literature: Cardiac Arrhythmia in the Premutation

Cardiac arrhythmia is not uncommon in premutation carriers especially in those who have FXTAS. Characteristics include atrial fibrillation (Greco et al., 2006), sick sinus syndrome (Greco et al., 2006), and unspecified cardiac arrhythmia (Hunsaker et al., 2011; Tassone et al., 2012a) have been reported. Many carriers who are elderly have significant bradycardia or other arrhythmia which requires a pacemaker (Greco et al., 2006; Hunsaker et al., 2011; Tassone et al., 2012a). There is only a rare report of arrhythmias in carriers without FXTAS including one case of a 7-years 6-month-old boy with an unmethylated 58 CGG repeats on *FMR1* gene who had multiple premature ventricular contractions (Tassone et al., 2000b). However, no direct study about cardiac arrhythmia in carriers of the premutation has been published.

### Case 2

Case 2 is a 60-year-old female with a *FMR1* premutation allele of 73 CGG repeats. Her father passed away at the age of 79 from FXTAS and congestive heart failure. Postural tremor in left hand started at age 49 followed by handwriting and memory problem at age 52 and decrease vibration sense in both feet at age 58. White matter lesions on brain magnetic resonance imaging (MRI) findings were consistent with FXTAS.

Mitral valve prolapse and regurgitation was detected when she was 53 years old. She also had orthostatic hypotension and autonomic instability when she was 57 years old. Hypertension has been noted since age 58. Tricuspid valve regurgitation, smaller left ventricle, and left ventricular outflow tract obstruction were also documented. She takes 25 mg of metoprolol for her cardiac conditions.

#### Review of Literature: Autonomic Dysfunction in the Premutation

Autonomic dysfunction is a common problem in the premutation carriers especially with FXTAS and it usually precedes diagnosis of FXTAS (Jacquemont et al., 2003, 2004; Pugliese et al., 2004; Leehey, 2009; Hagerman and Hagerman, 2013). In non-FXTAS carriers, a study found that the premutation females had reduced vagal tone which reflects impaired parasympathetic response (Klusek et al., 2017). In terms of cardiovascular disorders, the clinical presentation of autonomic dysfunction includes episodic hypotension (Pugliese et al., 2004; Greco et al., 2006; Gokden et al., 2009; Hagerman and Hagerman, 2013) and hypertension (Jacquemont et al., 2003; Coffey et al., 2008; Chonchaiya et al., 2010; Hamlin et al., 2012). Hypertension is the only condition which has been studied directly compared to controls and it is increased in carriers particularly those with FXTAS (Coffey et al., 2008; Hamlin et al., 2012).

Hypertension is highly prevalent in the premutation carriers particularly in the carriers with FXTAS. Since the Jacquemont et al. (2003) paper found that 50% of the premutation males had hypertension, there have been several studies which focused on prevalence of hypertension in premutation carriers. In females, a study found that 16.4% of premutation females without FXTAS had been diagnosed with hypertension compared with 10.1% in the age-matched control females (Coffey et al., 2008). The association was significant in females with FXTAS, where 61.1% had a history of hypertension compared with 18% of age-match control females (Coffey et al., 2008). In addition, hypertension tended to be more prevalent in premutation females who were daughters of males with FXTAS than the other carriers although there was no statistical significance (18.09 vs. 8.33%, respectively; *p* = 0.28) (Chonchaiya et al., 2010).

In premutation males, the results were consistent with findings in premutation females. A study of Hamlin et al. (2012) found that premutation males age 40 and above had 1.61 higher risk of having hypertension than controls. Their mean systolic blood pressure was 136.73 (SD 14.5) and diastolic blood pressure was 81.45 (SD 7.99) even on hypertensive medication. The risk of having hypertension was significant in the premutation males with FXTAS. They had an odds ratio 3.22 to have hypertension compare with age-matched control males. This study found that 67% of the premutation males with FXTAS and 41.5% of the premutation males without FXTAS had hypertension compare with 27.4% in the control group. Data about age of onset is still inconclusive since it was gathered retrospectively; however, it seemed comparable between the carriers and age-matched controls (Chonchaiya et al., 2010; Hamlin et al., 2012).

#### Review of Literature: Valvular Heart Disease in the Premutation

Valvular heart disease including mitral regurgitation and aortic stenosis in premutation carriers have been reported in individuals with FXTAS and some of them needed valvular replacement (Greco et al., 2006; Hunsaker et al., 2011).

### Case 3

A 41-year-old male with a *FMR1* premutation allele of 84 CGG repeats presented with a history of early connective tissue problems that included an umbilical hernia at 5 years old, recurrent bilateral muscle tears in the gastrocnemius muscles beginning at age 38, and spontaneous subluxation of the proximal interphalangeal joint of his right middle finger. Anxiety with panic attacks, migraine headaches, and obstructive sleep apnea has been described in his medical history. His daughter has the premutation and was the proband for the family.

At age 41, he was diagnosed with a dissection of the right upper cervical internal carotid artery extending into the proximal right petrous segment causing 60% luminal narrowing. He also had the right vertebral arterial dissection at C2 level, measuring 4 mm and a small left proximal cavernous internal carotid aneurysm. He did not undergo surgical intervention and was treated with aspirin 81 mg per day. Follow-up brain MRI was done 3 months after he was diagnosed which demonstrated significant resolution of the dissection and aneurysm.

### Review of Literature: Arterial Dissection and Aneurysm in the Premutation

This is the first report of internal carotid artery dissection, vertebral artery dissection, and internal carotid aneurysm in the premutation. Similarly, thoracic aortic dissection and abdominal aortic aneurysm have been reported as a cause of death in a 69-year-old male with FXTAS (Gokden et al., 2009). Recently, three premutation females with spontaneous coronary artery dissection (SCAD) who presented with chest pain have been documented (Park et al., 2017; McKenzie et al., 2020). These conditions have shared pathophysiology which is weakening arterial wall integrity from abnormal connective tissue morphology and inflammation (Hashimoto et al., 2006; Dogan et al., 2008; Sadasivan et al., 2013; Hayes et al., 2018; Yetkin and Ozturk, 2018).

## Discussion

**FIGURE 1:**
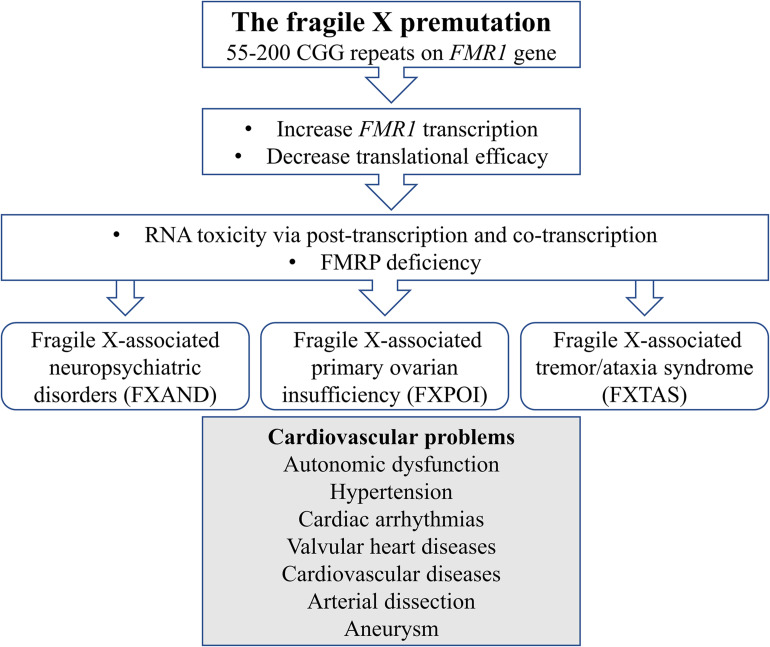
Schematic diagram illustrates mechanisms of cardiovascular problems in the fragile X premutation.

We report cases of atrial fibrillation, autonomic dysfunction, and vascular dissection in premutation carriers. To the best of our knowledge, this is the first perspective of cardiovascular problems in association with the fragile X premutation. We would like to postulate possible mechanisms for such an association: (1) RNA toxicity and (2) FMRP deficiency. They both play a role in each condition, but they are not mutually exclusive (Figure 1).

### The First Mechanism: RNA Toxicity

The concept of RNA toxicity has been reviewed extensively in FXTAS (Greco et al., 2006; Hagerman and Hagerman, 2013, 2016). In the premutation range, transcription of *FMR1* mRNA is enhanced compared to normal and the *FMR1* mRNA levels increase as the CGG repeat increases (Tassone et al., 2000a, 2007; Kenneson et al., 2001). Two mechanisms resulting from excessive mRNA are explained (Hagerman and Hagerman, 2015). The first mechanism is a post-transcriptional mechanism. The elevation of the mRNA sequestrates and binds to RNA binding proteins (i.e., DROSHA, DGCR8, Sam68, and HNRNPA2B1) and the sequestration of DROSHA/DGCR8 down regulates microRNA which are important for maintaining normal cellular functions (Garcia-Arocena and Hagerman, 2010; Sellier et al., 2010, 2013, 2014; Hagerman and Hagerman, 2013). The mRNA may also mistranslate because of RAN translation to FMRpolyG protein meaning FMRP with a polyglycine tail which is toxic to cells (Sellier et al., 2014, 2017). The second is a co-transcriptional mechanism. Increasing *FMR1* gene transcription links to elevation of R-loop formation and subsequently ineffective clearance of DNA damage repair responses (Loomis et al., 2014). These mechanisms cause mitochondrial dysfunction (Napoli et al., 2011; Song et al., 2016), disrupted intracellular calcium regulation (Robin et al., 2017), and eventually potentiate oxidative stress and inflammatory responses in the cells (Sellier et al., 2014; Hagerman and Hagerman, 2016). Elevated mRNA levels correlated with shorten neuronal survival and abnormal neuronal development in animal models with the *FMR1* premutation (Chen et al., 2010; Kaplan et al., 2012). Evidence of RNA toxicity and the sequestration of proteins is displayed as intranuclear inclusions in those with FXTAS which contain mRNA (Tassone et al., 2004) and the sequestered proteins (Iwahashi et al., 2006; Buijsen et al., 2014, 2016; Ma et al., 2019).

Other than in neuronal cells, the inclusions of FXTAS were also found in various organs including subepicardial autonomic ganglia, peripheral nervous system neurons, cardiomyocytes, and mitral valves (Greco et al., 2006; Gokden et al., 2009; Hunsaker et al., 2011; Buijsen et al., 2014) in addition to elevated mRNA levels (Greco et al., 2006; Hunsaker et al., 2011), therefore autonomic dysfunction which includes hypertension and cardiac arrhythmia are hypothesized to be a consequence of RNA toxicity. Increasing CGG repeats are also positively correlated with the numbers of inclusions (Greco et al., 2006). Moreover, anatomical and functional changes as well as findings of inclusion bodies in brain areas which regulate autonomic functions also support the hypothesis of RNA toxicity as a mechanism of autonomic dysfunction (Greco et al., 2006; Brown and Stanfield, 2015).

The mechanism of RNA toxicity may partly contribute to the explanation of cardiovascular conditions which link to connective tissue problems; these are arterial dissection (Hayes et al., 2018), aneurysm (Hashimoto et al., 2006; Sadasivan et al., 2013; Yetkin and Ozturk, 2018), and valvular heart disease (El Sabbagh et al., 2018). Increase inflammation and oxidative stress in carriers stimulated by RNA toxicity, have been thought to be an underlying pathophysiology in intracranial aneurysm (Hashimoto et al., 2006; Sadasivan et al., 2013; Yetkin and Ozturk, 2018) and arterial dissection (Hayes et al., 2018).

### The Second Mechanism: FMRP Deficiency

The mechanism might link to cardiovascular problems relate to connective tissue disorder in the carriers. The higher CGG repeats in carriers, especially more than 120 repeats, inversely correlate with FMRP levels (Pretto et al., 2014; Kim et al., 2019) which relates to some characteristics of connective tissue problems such as prominent ears (Riddle et al., 1998), elongated face, and hyperextensible finger joint (Loesch et al., 2003). The impact of FMRP deficiency on connective tissue has been well studied and reviewed in those with FXS (Ramírez-Cheyne et al., 2019). A pathological finding from a male with FXS showed abnormal structure and pattern of elastin, collagen, and acid mucopolysaccharide substrate in tissue samples from aorta, mitral and tricuspid valves, and forearm skin (Waldstein and Hagerman, 1988). From molecular studies, dysregulation of extracellular matrix-related proteins which are regulated by FMRP has been confirmed in FXS. These include matrix metalloproteinases (MMPs), elastin, and actin which are components of connective tissue structure (Ramírez-Cheyne et al., 2019).

The impact of FMRP deficiency on connective tissue in premutation carriers has not been studied. In respect to cardiovascular findings, diminished FMRP might affect endothelial integrity via alteration of MMP-9 expression which is hypothesized as a pathophysiology of dilating arteries (Hashimoto et al., 2006; Dogan et al., 2008; Sadasivan et al., 2013; Yetkin and Ozturk, 2018) and arterial dissection including SCAD (McKenzie et al., 2020). Moreover, the mechanism might lead to less elastic and stiffer vessels especially in those with a high CGG repeat range where FMRP is more deficient (Pretto et al., 2014).

Nonetheless, FMRP levels begin to be lower than average in individuals with a CGG above 120 (Pretto et al., 2014). RNA toxicity may also contribute to the connective tissue problems to some degree as a study found an association between some physical features of connective tissue disorder in the carriers who had normal FMRP level but high mRNA level (Basuta et al., 2011). Furthermore, intranuclear inclusions have been identified in mitral valve tissue from a carrier with mitral regurgitation who had high level of mRNA (Hunsaker et al., 2011). However, it is important to realize that mRNA and FMRP levels were measured in blood samples, so they might not represent actual levels in tissues (Tassone et al., 2000b) and need to be confirmed by direct studies.

### Future Directions

To better understand the risk of having cardiovascular problems in premutation carriers, more direct studies are needed in aspects of prevalence, age of onset, and mechanisms involved. For clinical implications, previous data currently proves that the premutation carriers have high risk of having hypertension (Coffey et al., 2008; Hamlin et al., 2012). Untreated hypertension not only contributes to atherosclerosis, but also causes white matter hyperintensity lesions in the brain which relate to impaired executive function, activities of daily living, gait speed, and mood (Hajjar et al., 2011). Therefore, it is very important to monitor blood pressure and treat as indicated.

In the aspect of cardiovascular diseases, chronic hypoestrogenism in the premutation females with FXPOI probably heightens risk of cardiovascular diseases via various mechanisms particularly atherosclerosis (Morselli et al., 2017). Early detection for FXPOI is crucial and the role of hormonal replacement therapy to reduce risk of having cardiovascular disease needs to be considered (Cobin et al., 2017). Nevertheless, research about the risk of having cardiovascular diseases in the premutation carriers has not been published. Even though ischemic heart disease and congestive heart failure have been reported in individuals with FXTAS (Jacquemont et al., 2003; Greco et al., 2006; Gokden et al., 2009), most of them are males and the mechanism is still unclear. Since FXTAS is associated with increasing mitochondria dysfunction (Napoli et al., 2011; Song et al., 2016), it is likely that end stage FXTAS is associated with such severe mitochondrial dysfunction that the congestive heart failure is also related to the lack of energy from mitochondrial problems (Brown et al., 2017; Zhou and Tian, 2018). Further study to confirm the association is necessary and a treatment for the mitochondrial dysfunction may eventually help myocardial function in aging carriers.

Finally, since neuropsychiatric disorders in carriers called FXAND are very common (Hagerman et al., 2018) and a long history of emotional intensity can lead to enhanced catecholamine release which could aggravate cardiovascular problems including cardiomyopathy (Wittstein et al., 2005), hypertension (Hamlin et al., 2012), and SCAD (McKenzie et al., 2020), therefore, treatment of FXAND should be kept in mind.

## Conclusion

The fragile X premutation carriers are likely to have health conditions throughout their life. This review underscores cardiovascular problems commonly seen in premutation carriers and alerts cardiologists and other clinicians to test for the premutation when necessary, watch for these problems and treat the conditions that arise related to the premutation.

## Data Availability Statement

The original contributions presented in the study are included in the article/supplementary material, further inquiries can be directed to the corresponding author/s.

## Ethics Statement

The studies involving human participants were reviewed and approved by Institutional Review Board at the University of California, Davis. The patients/participants provided their written informed consent to participate in this study. Written informed consent was obtained from the individual(s) for the publication of any potentially identifiable images or data included in this article.

## Author Contributions

NT, JC, RC, US, and RH discussed the manuscript content. JC, RC, and RH wrote case histories. NT wrote the first draft. All the authors reviewed and edited the manuscript before submission. All authors contributed to the article and approved the submitted version.

## Conflict of Interest

RH has consulted with Zynerba and Fulcrum regarding treatment of Fragile X syndrome and she has received funding from the Azrieli Foundation, Ovid, and Zynerba to carry out treatment studies in Fragile X syndrome. The remaining authors declare that the research was conducted in the absence of any commercial or financial relationships that could be construed as a potential conflict of interest.

## Abbreviations

ADHD: Attention-deficit/hyperactivity disorder
CGG: Cytosine-guanine-guanine
*FMR1*: Fragile X mental retardation 1
FMRP: Fragile X mental retardation protein
FXAND: Fragile X-associated neuropsychiatric disorders
FXPOI: Fragile X-associated primary ovarian insufficiency
FXS: Fragile X syndrome
FXTAS: Fragile X-associated tremor/ataxia syndrome
MMP: Matrix metalloproteinase
MRI: Magnetic resonance imaging
mRNA: Messenger RNA
SCAD: Spontaneous coronary artery dissection
SD: Standard deviation.
